# Identifying disrupted biological factors and patient-tailored interventions for chronic fatigue in adolescents and young adults with Q-Fever Fatigue Syndrome, Chronic Fatigue Syndrome and Juvenile Idiopathic Arthritis (QFS-study): study protocol for a randomized controlled trial with single-subject experimental case series design

**DOI:** 10.1186/s13063-022-06620-2

**Published:** 2022-08-19

**Authors:** Anouk Vroegindeweij, Joost F. Swart, Jan Houtveen, Niels Eijkelkamp, Elise M. van de Putte, Nico M. Wulffraat, Sanne L. Nijhof

**Affiliations:** 1 Department of Paediatric Rheumatology/Immunology, Wilhelmina Children’s Hospital, University Medical Center Utrecht, Utrecht University, Utrecht, The Netherlands; 2 Faculty of Medicine, Utrecht University, Utrecht, The Netherlands; 3 Center for Translational Immunology, University Medical Center Utrecht, Utrecht University, Utrecht, The Netherlands; 4 Department of Paediatrics, Wilhelmina Children’s Hospital, University Medical Center Utrecht, Utrecht University, Utrecht, the Netherlands

**Keywords:** Fatigue, Patient-tailored treatment, Randomized controlled trial, Experience sampling methodology, Q-fever fatigue syndrome, Chronic fatigue syndrome, Juvenile idiopathic arthritis, Lifestyle, Diet

## Abstract

**Background:**

Chronic fatigue with a debilitating effect on daily life is a frequently reported symptom among adolescents and young adults with a history of Q-fever infection (QFS). Persisting fatigue after infection may have a biological origin with psychological and social factors contributing to the disease phenotype. This is consistent with the biopsychosocial framework, which considers fatigue to be the result of a complex interaction between biological, psychological, and social factors. In line, similar manifestations of chronic fatigue are observed in chronic fatigue syndrome/myalgic encephalomyelitis (CFS/ME) and juvenile idiopathic arthritis (JIA). Cognitive behavioral therapy is often recommended as treatment for chronic fatigue, considering its effectiveness on the group level. However, not everybody benefits on the individual level. More treatment success at the individual level might be achieved with patient-tailored treatments that incorporate the biopsychosocial framework.

**Methods:**

In addition to biological assessments of blood, stool, saliva, and hair, the QFS-study consists of a randomized controlled trial (RCT) in which a single-subject experimental case series (*N=1*) design will be implemented using Experience Sampling Methodology in fatigued adolescents and young adults with QFS, CFS/ME, and JIA (aged 12–29). With the RCT design, the effectiveness of patient-tailored PROfeel lifestyle advices will be compared against generic dietary advices in reducing fatigue severity at the group level. Pre-post analyses will be conducted to determine relevance of intervention order. By means of the *N*=1 design, effectiveness of both advices will be measured at the individual level.

**Discussion:**

The QFS-study is a comprehensive study exploring disrupted biological factors and patient-tailored lifestyle advices as intervention in adolescent and young adults with QFS and similar manifestations of chronic fatigue. Practical or operational issues are expected during the study, but can be overcome through innovative study design, statistical approaches, and recruitment strategies. Ultimately, the study aims to contribute to biological research and (personalized) treatment in QFS and similar manifestations of chronic fatigue.

**Trial registration:**

Trial NL8789. Registered July 21, 2020.

**Supplementary Information:**

The online version contains supplementary material available at 10.1186/s13063-022-06620-2.

## Administrative information

Note: the numbers in curly brackets in this protocol refer to SPIRIT checklist item numbers. The order of the items has been modified to group similar items (see https://www.equator-network.org/reporting-guidelines/spirit-2013-statement-defining-standard-protocol-items-for-clinical-trials/)

**Table:** 

Title {1}	Identifying disrupted biological factors and patient-tailored interventions for chronic fatigue in adolescents and young adults with Q-Fever Fatigue Syndrome, Chronic Fatigue Syndrome and Juvenile Idiopathic Arthritis (QFS-study): study protocol for a randomized controlled trial with single-subject experimental case series design
Trial registration {2a and 2b}.	Trial NL8789 (www.trialregister.nl), registered July 21, 2020.
Protocol version {3}	March 25^th^ 2021, version 6
Funding {4}	Our study is granted by the Netherlands Organization for Health Research and Development (ID ZonMW 50-53000-98-566) and received additional funding through crowdfunding.
Author details {5a}	Anouk Vroegindeweij^a^; Sanne L. Nijhof^b,c^; Joost. F. Swart^a,c^; Jan Houtveen^a^; N. Eijkelkamp^d^; Elise M. van de Putte^b,c^; Nico M. Wulffraat^a,c^ ^a^ Department of Paediatric Rheumatology/Immunology, Wilhelmina Children’s Hospital, University Medical Center Utrecht, Utrecht University, Utrecht, The Netherlands ^b^ Department of Paediatrics, Wilhelmina Children's Hospital, University Medical Center Utrecht, Utrecht University, Utrecht, the Netherlands ^c^ Faculty of Medicine, Utrecht University, Utrecht, The Netherlands ^d^ Center for Translational Immunology, University Medical Center Utrecht, Utrecht University, Utrecht, The Netherlands
Name and contact information for the trial sponsor {5b}	University Medical Center Utrecht. Address: Heidelberglaan 100, 3584CX Utrecht, The Netherlands. Phone: 088-75 555 55. E-mail: info@umcutrecht.nl
Role of sponsor {5c}	The content is the responsibility of the authors and does not necessarily represent the official views of University Medical Center Utrecht. The funding body ZonMW and crowdfunding had no role either in the study design, data collection and analysis, preparation of the manuscript, or decision to publish.

## Introduction

### Background and rationale {6a}

Q-fever is a zoonosis caused by the intracellular gram-negative bacterium *Coxiella burnetii*. Severe chronic fatigue is a frequently reported complaint among patients with a history of Q-fever infection and is described as persistent or recurrent severe fatigue with a profound, debilitating effect on daily life [1, 2]. Especially in children, chronic fatigue affects future well-being and social participation [2, 3]. The most recent Q-fever epidemic took place in The Netherlands from 2007 to 2010 [4]. Since then, up to a dozen of new cases of Q-fever have been confirmed every year [5, 6]. Most of those affected with acute Q-fever recover from fatigue within 6 to 12 months [4]. Approximately 20% does not recover within this timeframe, leading to Q-fever fatigue syndrome (QFS) diagnosis [4, 7]. QFS has been associated with immune deregulation [8] and possibly mitochondrial dysfunction [9]; however, the etiology and pathophysiology of QFS is still largely unknown [8, 9].

The term QFS is derived from chronic fatigue syndrome (CFS), also known as myalgic encephalomyelitis (ME), because of its similar expression. Like with CFS/ME, individuals with QFS have, in addition to severe fatigue, a complex set of symptoms including, e.g., headache, muscle and/or joint strains, sore throat, and painful lymph nodes [6, 7, 10]. Post exertional malaise, unrefreshing sleep, memory or concentration difficulties, and issues regarding mental well-being, are also common [6, 7, 10]. As a result, daily functioning and participation in society are significantly limited [6, 7, 10].

An overlap in symptoms similar to QFS and CFS/ME is also observed in individuals suffering from fatigue in (auto-)immune diseases, such as juvenile idiopathic arthritis (JIA). Previous research has shown that severe fatigue is prevalent among 25% of individuals with pediatric rheumatic diseases [11], which is more prevalent than in the general population [2]. One systematic review reported fatigue among 60–75% of all JIA cases [12], and fatigue has been identified as a long-term sequela in 26% of JIA cases [13].

Fatigue is conceptualized as a generic symptom across diseases, instead of a disease-specific symptom [14]. Enck et al. hypothesized that medically unexplained functional symptoms following infection, such as fatigue, may have a biological origin, with psychological and social factors contributing to the disease phenotype [15]. This is consistent with the theoretical framework of the biopsychosocial model, in which fatigue can be seen as the result of a complex interaction between biological, psychological, and social factors [16, 17].

Even without knowing the exact primary biological events and subsequent cascades, the most promising treatment options for chronic fatigue are based on cognitive behavioural interventions (CBT) addressing dysfunctional cognitions, affect, physical activity, social support, or sleep patterns [18, 19]. Although CBT is effective on the group level [18, 19], it is not successful in all individuals. With regard to QFS, one study in adults showed positive effects on fatigue directly after CBT across subjects [20]—but without lasting effect for the majority at 1-year follow-up [21]. Further investigation on tailoring treatment to QFS was recommended [21].

We expect that it is even more effective to tailor treatment at the individual level, whilst incorporating the biopsychosocial perspective. This can be achieved by using experience sampling methodology (ESM), a method in which individuals receive multiple surveys per day for a longer period of time, resulting in the collection of highly intensive longitudinal data (ILD) [22]. ESM allows dynamic assessment of individual fatigue-perpetuating biological, psychological, and/or sociological factors, and has been implemented in PROfeel. PROfeel is a smartphone-based ESM method, followed by individual symptom network analysis, of which the results are used as starting point for face-to-face feedback and shared decision-making with the patient regarding lifestyle advice. The PROfeel method has been found feasible and useful by fatigued adolescents [23]. The next step is to test whether patient-tailored lifestyle advices based on the PROfeel method are also effective in reducing fatigue severity in the QFS-study.

The patient-tailored PROfeel lifestyle advices will be compared to generic dietary advices as potential treatment options. Nutritional advice has been effective in reducing medically unresolved fatigue symptoms in children before [24]. Based on an established relationship between the gut microbiome and fatigue, it is expected that a healthy diet positively influences the gut microbiome, potentially reducing fatigue [25].

In this paper, we describe the study objectives and methods of the QFS-study. The intervention part of the study consists of two designs. First, a randomized controlled trial (RCT), to compare the effectiveness of patient-tailored PROfeel lifestyle advices against generic dietary advices in reducing fatigue severity in adolescents and young adults with QFS, CFS/ME, and JIA at the group level. Second, a single-subject experimental case series (i.e., *N*=1) design to test the effectiveness of both advices at the individual level. The *N*=1 design will be implemented with PROfeel. Considering that the etiology and pathology of QFS is largely unknown, the QFS-study also includes biological research. Multiple biological assessments will be conducted to identify disrupted biological factors in participants with QFS, in comparison to participants with CFS/ME and JIA, or healthy controls.

### Objectives {7}

The first objective of the current study is to evaluate the effectiveness of patient-tailored PROfeel lifestyle advices and generic dietary advices as interventions on “fatigue severity”, “quality of life”, and “self-efficacy” on the group level. The second objective is to explore the same, but on the individual level for all participants (i.e., multiple *N*=1). If patient-tailored PROfeel lifestyle advices are effective, the third objective is to investigate through which mechanisms the effects have been reached, by exploring changes in the dynamic ESM networks pre-post intervention. The fourth objective is to explore the biological profile (e.g., immunological profile, HPA axis, mitochondrial dysfunction, and gut microbiome) of adolescents and young adults with QFS at baseline. Participants with QFS will be compared to participants with CFS/ME and JIA, as well as healthy controls. The fifth objective is to explore relationships between individual improvement differences in “fatigue severity” and subsequent changes in biological profile.

### Trial design {8}

The intervention part of the QFS-study consists of two designs. First, a RCT with concealed block randomization and 1:1 allocation ratio. Second, an exploratory single-subject experimental case series design for all participants (i.e., multiple *N*=1). Biological assessments will be completed at three out of four RCT study visits.

## Methods: participants, interventions, and outcomes

### Study setting {9}

The QFS-study will recruit 60 fatigued participants for the RCT (3 subgroups: QFS, CFS/ME, and JIA, each *N*=20), and up to 80 healthy controls for the biological objectives. Recruitment will take place at the Wilhelmina Children’s Hospital, part of the University Medical Centre Utrecht (UMCU) in Utrecht, The Netherlands.

### Eligibility criteria {10}

To be eligible, fatigued participants and healthy controls must be at least 12 years old and no older than 29 years during baseline. All must be able to speak, read, understand, and write Dutch. Fatigued participants must own a smartphone. Individuals with a diagnosis of chronic Q-fever and active disease are excluded from participation. Individuals are also excluded in case of cognitive impairment with an estimated IQ of below 70, or any concomitant (predominant psychiatric) diagnosis that can explain fatigue at baseline, such as major depressive disorder.

#### Additional criteria for participants with QFS

All 20 participants must enter the screening process with a (suspected) diagnosis of QFS according to the Dutch guidelines [25]. Participants must have suffered from debilitating fatigue with detrimental effects on daily functioning in work and/or private life for at least 6 months. The debilitating fatigue must have started before the age of 18, and after infection with *Coxiella burnetii*—where fatigue severity increased significantly after infection. All participants in the QFS group must be seropositive for *Coxiella burnetii* and cannot have a diagnosis of chronic Q-fever or recent diagnostics (< 3 months) demonstrating an IgG phase 1 titer of <1:1024 (or 1:512 in case of patients who are immunocompromised, or with a vascular prosthesis or a heart defect). At the time of screening, fatigue must be severe. This is indicated by a score higher than 39 on the Checklist Individual Strength-8 (CIS-8) questionnaire, which ranges from 0 to 56, with higher scores indicating more fatigue [26].

#### Additional criteria for participants with CFS/ME

All 20 participants must enter the screening process with a (suspected) diagnosis of CFS/ME according to the Centers for Disease Control (CDC) criteria [1]. The biological trigger for CFS/ME can be unknown or diverse. Participants with SARS-COV-2 infection prior to CFS/ME diagnosis with CDC criteria are not excluded. Participants cannot have a QFS diagnosis. At the time of screening, fatigue must be severe (i.e., CIS-8 > 39).

#### Additional criteria for participants with JIA

All 20 participants must enter the screening process with a diagnosis of JIA. Participants must be on stable medication and have a stable disease activity score (Juvenile Arthritis Disease Activity Score; JADAS criteria) [27] for at least 3 months. Participants cannot have a QFS diagnosis. At the time of screening, the participant must express fatigue as a main complaint in the last 3 months. At the time of screening, fatigue must be severe (i.e., CIS-8 > 34, which indicates severe fatigue in JIA).

#### Additional criteria for healthy controls

Healthy controls must have no severe fatigue complaints (i.e., CIS-8 ≤ 39) and may either be seropositive or seronegative for *Coxiella burnetii* or SARS-COV-2. Serological status will be assessed after inclusion. Healthy controls are preferably siblings or friends of participating patients with QFS to increase the likelihood of recruiting healthy individuals with seropositive *Coxiella burnetii* status.

### Who will take informed consent? {26a}

The executive investigator will provide participants with oral and written information about the QFS-study, and obtain written informed consent from all participants before participation. In case participants are younger than 16 years, written informed consent will also be obtained from both parents or legal guardians before participation.

### Additional consent provisions for collection and use of participant data and biological specimens {26b}

Before participation in the study, the executive investigator will obtain written informed consent for the collection, storage and analysis of participant data, and biological specimens (i.e., blood, stool, saliva, and hair samples) from all participants. In case participants are younger than 16 years, written informed consent will also be obtained from both parents or legal guardians before participation.

## Interventions

### Explanation for the choice of comparators {6b}

The effectiveness of patient-tailored PROfeel lifestyle advices in reducing “fatigue severity” and improving “quality of life” and “self-efficacy” will be compared with generic dietary advices based on the Netherlands Nutrition Centre (NNC) (www.voedingscentrum.nl). The PROfeel method has previously been found feasible and useful by fatigued adolescents [23]. The next step is to test whether the patient-tailored PROfeel lifestyle advices are also effective in terms of reducing “fatigue severity”. Based on an established relationship between the gut microbiome and fatigue [28], a healthy diet is expected to positively influence the gut microbiome, potentially reducing fatigue. It has been shown before that nutritional advice can decrease some medically unresolved fatigue complaints in Dutch children [24].

### Intervention description {11a}

#### Patient-tailored PROfeel lifestyle advice

The RCT starts with a 4-week ESM assessment (see Table 1 for timeline). ESM is an innovative, validated, structured diary technique for longitudinal measurements of fluctuating states, that captures life as it is lived on the spot [22, 23, 29]. Multiple ESM applications are available for smartphones. In this study, we use the Ethica application (www.ethicadata.com) in which participants complete ESM surveys (i.e., diaries) approximately 5 times per day. In a first step, ESM data are used by a statistician to compute an individual descriptive report and an individual dynamic report with fatigue-perpetuating dynamic networks, identifying the contemporaneous and time-lag relations between fluctuations in fatigue and behavior, sleep, social context, cognitions, and feelings [23]. In a next step, the investigator uses the descriptive and dynamic reports to produce a reduced PROfeel report. Face-to-face, the investigator and participant discuss the PROfeel report and use shared decision-making to formulate a patient-tailored lifestyle advice. The term “lifestyle” here refers to an area of daily life that can be modified to potentially reduce fatigue and/or improve quality of life and self-efficacy. The combination of ESM with personalized feedback, leading to patient-tailored lifestyle advices, is named PROfeel. Details follow in the sections underneath.

**Table 1:** Participant timeline in QFS-study

Week	Activity	Description
0	Screening	Evaluation of participant’s eligibility for the QFS-study by social pediatrician (and pediatric immunologist/rheumatologist in case of JIA diagnosis).
1	Study visit T0	Participant completes baseline KLIK questionnaires and installs the PROfeel app on smartphone to start ESM measurement. Participant also starts weekly measurement (of fatigue, quality of life, and self-efficacy) on smartphone which lasts throughout the study.
1-4	ESM phase I	Participant starts 4 weeks of ESM, with 5 surveys to answer per day. Surveys contain items inquiring after, e.g., fatigue and other symptoms, (social) activities, and affect in the last 3 h.
5	Study visit T1	Participant completes T1 KLIK questionnaires and is randomly allocated to either 12 weeks of patient-tailored PROfeel lifestyle advice or generic dietary advice. Biological samples (i.e., blood, stool, saliva, and hair) are collected for the first time.
5-16	Intervention I	Participant adheres to allocated advice for 12 weeks. To improve adherence, participant receives a boost from the investigator by phone and/or mail.
17	Study visit T2	Participant completes T2 KLIK questionnaires and evaluates first intervention with investigator. Biological samples (i.e., blood, stool, saliva, and hair) are collected for the second time.
17-20	ESM phase II	Participant starts a second period of 4 ESM weeks, with 5 surveys to answer per day. Survey items are identical to ESM phase I.
21	Study visit T3	Participant completes T3 KLIK questionnaires and is allocated to the other intervention arm (either 12 weeks of patient-tailored PROfeel lifestyle advice or generic dietary advice).
21-32	Intervention II	Participant adheres to allocated advice for 12 weeks. To improve adherence, participant receives a boost from the investigator by phone and/or mail.
33	Study visit T4	Participant completes T4 KLIK questionnaires and evaluates second intervention with investigator. If fatigue remains severe, participant can be referred to ECCF for post-trial care. Biological samples (i.e., blood, stool, saliva, and hair) are collected for the third time.
33-60	Follow-up	Weekly measurement (of fatigue, quality of life, and self-efficacy) on smartphone continues until 12 weeks after T4. During week 60, participant completes follow-up KLIK questionnaires, marking the end of QFS-study participation.

Healthy controls only have one study visit during which questionnaires (i.e., baseline KLIK, Eetscore, and optionally Food Frequency Questionnaire) and biological samples (i.e., blood, stool, saliva, and hair) are collected

*ESM* experience **s**ampling methodology, *CIS-8* Checklist Individual Strength-8 questionnaire, *ECCF* Dutch Expert Centre for Chronic Fatigue

#### PROfeel: ESM assessment

The ESM surveys are structured within the framework of the biopsychosocial model [16, 17], meaning that all surveys consist of a fixed set of items regarding potentially fatigue-perpetuating factors of biological, psychological, and social nature. These factors are part of the theoretical framework of efficacious elements of CBT [18]. Next to the fixed set, participants can add personalized items of their choice. These items represent additional somatic symptoms, symptom-related behaviors, feelings and cognitions, and a self-selected factor. This way, participants receive personalized surveys. Depending on the personalization, the surveys consist of 12 to 21 items. All items (e.g., “in the last 3 hours, I was fatigued”) are answered on a visual analogue scale (VAS) scale ranging from “not at all” to “very much”. Completing the personalized survey takes approximately 1 min. The ESM content was developed in an interdisciplinary team of researchers and clinicians and was previously tested in a feasibility study [23].

With regard to compliance, participants are asked to respond to at least 100 out of 140 ESM surveys (i.e., ≥70% compliance) to allow the computation of a reliable descriptive report and a reliable dynamic report with fatigue-perpetuating dynamic networks. PROfeel uses a time-based ESM recording method with interval contingency, meaning that all surveys are distributed at a fixed interval of 3 h. To allow for the highest compliance, participants are asked during the intake to select at what time slots they would like to receive their surveys (e.g., starting at 9 AM, 10 AM, or 11 AM each day).

#### PROfeel: feedback report and shared decision-making

After the ESM assessment, the investigator and statistician independently select which figures from the descriptive and dynamic reports should be discussed with the participant. This selection is based on statistical significance and relevance (i.e., weight and context of the dynamic network factor loadings, potential contribution to the patient’s self-knowledge, and usefulness as input for patient-tailored PROfeel lifestyle advice) and compiled into a PROfeel report. During a face-to-face meeting, the investigator discusses the PROfeel report with the participant. Input from the participant is leading for the interpretation of the figures. Participants are always asked to indicate the extent to which they recognize themselves in each figure. Two example figures are shown in the Additional files 1 and 2 (Figs. 1 and 2). Patients have considered the visual presentation of their symptoms a strong feature of PROfeel [23].

Once the PROfeel report is discussed, the investigator and participant decide what type of personalized lifestyle advice might help to reduce fatigue severity and/or improve quality of life and self-efficacy in daily life. This advice can be based directly on the figures from the PROfeel report, but may also be linked to something that came up during the meeting. Thus, the PROfeel report is used as a clinical conversation tool to create patient-tailored lifestyle advices. The report is not intended to be the sole input for lifestyle advices.

#### PROfeel: defining patient-tailored lifestyle advice

During shared decision-making, the investigator helps to define the lifestyle advice as feasible as possible. This is important since lifestyle advices often revolve around habits that need to be (re)learned. Because changing or learning habits is a slow process [30], the patient-tailored PROfeel lifestyle advices often represent a step in the participant’s process of (re)learning a habit. The advices incorporate factors known to strengthen habit learning, such as anticipating and avoiding self-control struggles, planning, and/or environment changes [30]. For example, when a participant struggles with rumination (i.e., worrying) throughout the day, an advice could be to reduce rumination time by practicing distracting attention. The investigator and participant could decide to work with a patient-tailored variation of “scheduled rumination” [31], in which the participant chooses a fixed timeslot to ruminate for 15 min, for instance, every day from 3:00 AM to 3:15 AM. To prevent self-control struggles, the participant and investigator compile a list of activities that help distract the participant from rumination. Whenever the participant finds him/herself ruminating outside the scheduled time, he/she is instructed to notice the thoughts, write them down if necessary, and divert attention with an activity on the list. Practicing scheduled rumination may lead to short-term improvement, provide insight in what the next step should be, and motivate the participant to take that next step in the long term.

Eventually, the patient-tailored PROfeel lifestyle advices will be adhered to for 12 weeks. During each week, the participant will receive a weekly survey on the Ethica app, inquiring about the levels of fatigue, quality of life, and self-efficacy in the past week, and whether he or she complied with the patient-tailored lifestyle advices.

#### Dietary advices

After the ESM assessment, the participant starts with either patient-tailored PROfeel lifestyle advices or generic dietary advices (see Table 1). The dietary advices are based on healthy and sustainable food-based dietary guidelines from the Netherlands Nutrition Centre (NNC), adjusted for age and gender [32]. The investigator provides the participant with information on the NNC dietary recommendations, including an overview of recommended foods and drinks available in local supermarkets. The general Dutch population is familiar with these guidelines as the “Wheel of Five”, a nutritional-counselling model that helps Dutch consumers make healthy food choices. In addition, participants fill out the “Eetscore”, an instrument developed to help consumers and health professionals improve their eating habits [33]. The instrument assesses the extent to which food and beverage intake conforms to the NNC’s dietary recommendations. The Eetscore provides 16 subscores, with higher scores indicating better adherence to the guidelines. The scores are presented as feedback to the participant, accompanied by educational background information on the guidelines and tips and tricks for incorporating the recommendations in daily life. Through the Eetscore tool, personal areas for dietary improvement are highlighted to the participant.

The participant adheres to the diet for 12 weeks. During these 12 weeks, the participant receives two weekly surveys. One survey requiring after the levels of fatigue, quality of life, and self-efficacy, and one survey requiring after the level of compliance to each dietary guideline. When participants respond non-adherence to a guideline, they will be asked to indicate whether they consumed more or less than recommended. This results in a weekly nutrition survey of 16 to 27 items.

### Criteria for discontinuing or modifying allocated interventions {11b}

This is a minimal risk study. Therefore, we have not specified criteria for discontinuing or modifying allocated interventions. Study participation is voluntary. Participants always have the right to withdraw from participation prematurely at any time, without providing an explanation. The investigator may also withdraw any participant who is unable to complete the QFS-study due to deteriorating health or another reason.

### Strategies to improve adherence to interventions {11c}

During the RCT, participants receive a boost from the investigator by phone and/or mail. Participants are also regularly reminded that they can contact the investigator for questions or help with their advice or technical problems with the app.

To improve adherence to the patient-tailored PROfeel lifestyle advices, the advices are formulated clearly and concretely (e.g., “cycle to school 3 days a week to increase physical activity” rather than “increase physical activity”). This way, participants know what is expected of them. Participants receive a weekly survey item on their smartphone, inquiring after their level of compliance.

To improve adherence to the generic dietary advice, participants receive a weekly survey inquiring after their compliance to each aspect of the dietary advice. The surveys function as both monitoring and reminder tools for the participant. Reminders are often used as a tool in behavior change interventions [30], and a meta-analysis has shown that reminders can make a small contribution to behavior change in dietary interventions [34].

### Relevant concomitant care permitted or prohibited during the trial {11d}

Participants are ineligible if they already participate in a behavioral intervention aimed at reducing fatigue severity. However, it is allowed to receive concomitant care focused at reducing fatigue severity if this is part of the patient-tailored PROfeel lifestyle advice (e.g., physiotherapy directed at body mentalization). It is also allowed to receive concomitant medical or psychological care, if this was already started before the intervention and as long as the primary focus of this treatment is not directed at reducing the fatigue.

### Provisions for post-trial care {30}

Before enrollment in the RCT, all fatigued participants will receive oral and written information of optional post-trial care at the Dutch Expert Centre for Chronic Fatigue (ECCF). Regular care, such as CBT will be offered following the study without waiting list. If desired, the participant decides in consultation with the therapist whether this treatment will be followed additionally.

### Outcomes {12}

Primary outcome of the intervention study is “fatigue severity”. Secondary outcomes are “self-efficacy” and “quality of life”. All outcomes are measured at study visits T0–T4 and through weekly measurement by smartphone (see Table 1). For details on outcome measurement, see section “Plans for assessment and collection of outcomes” {SPIRIT Item 18a}. For information on the biological study outcomes and laboratory evaluations, see section “Plans for collection, laboratory evaluation, and storage of biological specimens for genetic or molecular analysis in this trial/future use” {SPIRIT Item 33}.

### Participant timeline {13}

Participant timeline is shown in Table 1.

### Sample size {14}

As described earlier, the QFS-study concerns a RCT with implemented *N*=1 design. The objectives of the study with *N*=1 design do not require a sample size calculation. Therefore, power analysis was performed for the group-level analyses on the primary outcome “fatigue severity”, which will be measured pre-post interventions. Based on previous CBT studies aiming to reduce chronic fatigue [18, 26], with an alpha of .05, power of .80, repeated measures correlation of .23, and an anticipated Cohen’s *f* effect size of .5, the sample size calculation for repeated measures 2 × 2 ANOVA yields *n*=16 per group. Taking into consideration an expected drop-out rate of 20% from previous single-subject experimental case research with multiple measurement on severe fatigue in adolescent patients with an immune dysregulation disorder, we will include 20 participants per group (i.e., QFS, CFS/ME, JIA), resulting in *N*=60. With *N*=60, it was decided upon expert medical and research lab opinion to include up to 80 healthy controls to increase the likelihood of recruiting healthy controls with seropositive *Coxiella burnetii* status, of observing contrasts in biological parameters, and to enable matching for age and gender as much as possible.

### Recruitment {15}

Adolescents and young adults with QFS and long-COVID will be notified through respectively the Q-support network and C-support network, as well as through notifications on the UMC Utrecht and University of Utrecht (UU) website. Adolescents with CFS/ME and JIA are regularly seen at the outpatient clinics by general pediatricians and pediatric immunologist/rheumatologists at UMC Utrecht and made aware of the study through them. After a uniform diagnostic work-up (via medical history, laboratory examinations, and questionnaires) done by the social pediatrician and pediatric immunologist/rheumatologists, patients and their parents receive detailed oral and written information about the QFS-study from the investigator. Information about the study has been approved by the Institutional Review Board (IRB) of UMC Utrecht (reference NL72103.41.20, IRB 20/166). Ultimately, all adolescents and young adults complying with the defined inclusion criteria will be invited for this study.

## Assignment of interventions: allocation

### Sequence generation {16a}

Fatigued participants will be randomized to their first intervention, which is either patient-tailored PROfeel lifestyle advices or generic dietary advice, in a 1:1 ratio. Computer-generated concealed block randomization will be used. Randomization will be stratified by group (i.e., QFS, CFS/ME, and JIA) to distribute participants equally across the intervention arms.

### Concealment mechanism {16b}

Concealment will be accomplished electronically. Only one independent statistician has access to the randomization outcome. In case the participant will be assigned to the patient-tailored PROfeel lifestyle condition, it is necessary for the investigator to know this in advance in order to prepare the PROfeel report. Therefore, the investigator contacts the statistician by email to inquire about the allocation for that participant, three working days before the second study visit (i.e., T1).

### Implementation {16c}

The allocation sequence was generated in SPSS by the independent statistician on the research team and he is the only one with access to the randomization outcome. The investigator is responsible for contacting the statistician three working days before the second study visit (T1) to inquire allocation. The statistician responds by email. In all correspondence, the investigator and statistician use pseudonymized codes to refer to participants (e.g., “QFS-1”, CFS-3”, and “JIA-20”).

## Assignment of interventions: blinding

### Who will be blinded {17a}

The analyses of the ESM data will be conducted by the independent statistician, who has no contact with the participants. In order for the statistician to interpret the ESM data without prior knowledge of the participant, the investigator will only refer to participants using an anonymous code (e.g., “QFS-1”, “CFS-3”, and “JIA-20”). When the statistician reveals the assignment to the first condition, the investigator will share this with the participant during study visit T1.

All biological samples (i.e., blood, stool, saliva, hair) will be labelled with the participant’s study number without any reference to diagnosis (i.e., 1, 2, … 140). Only the investigator has an overview of which study number matches which participant. The investigator is responsible for matching healthy controls to fatigued participants as much as possible, based on age and gender. The investigator is not present during lab analyses.

### Procedure for unblinding if needed {17b}

No procedures are needed for unblinding.

## Data collection and management

### Plans for assessment and collection of outcomes {18a}

The primary outcome of the intervention study is “fatigue severity” as measured by the subscale “fatigue severity” (8 items) of the Checklist Individual Strength-20 (CIS-20). The subscale consists of 8 items (CIS-8). Participants will receive the CIS-8 once a week throughout the study as a survey in the Ethica smartphone app. The weekly CIS-8 has a scoring range of 8–56, with higher scores indicating more fatigue. The CIS-8 has good reliability and discriminative validity [35]. A reduction of 1 standard deviation (SD) is considered clinically relevant.

Secondary outcomes of the intervention study are “quality of life” and “self-efficacy”. Per construct, one VAS item will be added to the weekly CIS-8 questionnaire. In addition, these outcomes will be measured with questionnaires in KLIK, a web-based survey portal (www.hetklikt.nu). “Quality of life” will be measured with the Pediatric Quality of Life Inventory 4.0 Generic Score (PedsQL GCS). This questionnaire consists of four subscales (i.e., physical, emotional, social, and school or work functioning), with total- and subscores ranging from 0 to 100. Higher scores indicate a higher quality of life. The PedsQL GCS has good validity and reliability [36]. An increase of 1 SD is considered clinically relevant. “Self-efficacy” is defined as a sense of control over fatigue symptoms and will be measured with the Self-Efficacy Scale-28 (SES-28). This questionnaire consists of 5 items that inquire about the sense of control related to fatigue, with higher scores indicating more sense of control [37]. An increase of 1 SD will be considered clinically relevant.

With KLIK, we also measure possible predictors, mediators, or moderators of the primary outcome “fatigue severity”. The choice of measurements was guided by whether the following aspects could be predictive for the effectiveness of the interventions. The outcomes are: “fatigue severity at baseline” measured with the CIS-8 questionnaire [26], “quality of life at baseline” measured with the PedsQL GCS [36], “self-efficacy at baseline” measured with the SES-28 [35], “sleep problems” measured with the PedsQL Multidimensional Fatigue Scale (PedsQL MFS) [36], “daily participation” measured by school or work presence expressed in hours attended divided by hours obliged multiplied by 100, “pain level” measured using a visual analogue scale (VAS) to indicate average pain over the last week, and “depression or anxiety symptoms” measured with the Revised Child Anxiety and Depression Scale (RCADS) [38].

KLIK measurements are completed at baseline (T0), pre-intervention (T1 and T3), and post-intervention (T2 and T4). See also the participant timeline in Table 1.

With the Eetscore tool, we measure dietary intake pre- and post-dietary intervention. The outcome is the total difference score, which reflects the amount of change in dietary intake as a result of the dietary intervention. The Eetscore is typically used for assessing dietary intake in people aged 18 years or older [33]. For the QFS-study, the University of Wageningen adjusted the Eetscore to make it usable for people aged 12 years and older. To enable validation of the adjustments, participants below the age of 18 will be given the option to also complete the validated Food Frequency Questionnaire (FFQ) [39].

Regarding the biological outcomes and laboratory evaluations, see section “Plans for collection, laboratory evaluation, and storage of biological specimens for genetic or molecular analysis in this trial/future use {33}”.

### Plans to promote participant retention and complete follow-up {18b}

The QFS-study will use multiple strategies to promote that participants do not drop out of the study and complete follow-up. For example, we will minimize the burden of assessments, send reminder letters, emails, or phone calls, provide patients with study team contact information, and combine study visits with usual care visits whenever possible. Study visits will be scheduled as flexible as possible. To enable intention-to-treat analyses, participants who withdraw prematurely from the QFS-study will be encouraged to complete follow-up assessments. Participants’ reasons for withdrawing will be recorded whenever possible.

### Data management {19}

Collected data will be stored by the Wilhelmina Children’s Hospital of UMC Utrecht for 15 years following completion of the project. A Data Management Plan (DMP) has been prepared and evaluated by the UMC Utrecht’s data quality management team. It applies FAIR (Findable, Accessible, Interoperable, Reusable) principles to the QFS-study data.

### Confidentiality {27}

Data will be handled confidentially according to the rules of General Data Protection Regulation (GDPR). Only researchers directly involved in the project will have access to the codes and participant data, which will only be stored in password-protected files. However, in case participants decide to follow post-trial care at the ECCF, the treating therapist at the ECCF will receive the baseline assessment and PROfeel data required to indicate which treatment modules should be provided. Computers used to access the data will be protected in accordance with UMC Utrecht standards. Paper forms (e.g., informed consent from participants, parents or legal guardians, and investigator notes from PROfeel conversations) will be stored in a locked file cabinet at the Wilhelmina Children’s Hospital.

### Plans for collection, laboratory evaluation, and storage of biological specimens for genetic or molecular analysis in this trial/future use {33}

In the QFS-study, biological samples will be collected from participants at study visits T1, T2, and T4 (see Table 1). Healthy controls will have only one study visit, during which the same biological samples will be collected (i.e., blood, stool, saliva, and hair). All biological samples will be stored and saved at the UMC Utrecht biobank for 15 years.

The T1 samples will be used to explore the biological profile and to identify disrupted biological factors in fatigued participants (objective 4). Participants will be matched to healthy controls, based on age and gender. Blood samples will be used to (1) determine the sensitivity of the immune system to regulation by glucocorticoids in participants compared to Q-fever seropositive and Q-fever seronegative healthy and controls; (2) assess the sensitivity to adrenergic regulation of the immune system in QFS, CFS/ME, and fatigued JIA participants compared to respective healthy controls and; (3) to assess metabolic dysfunction by means of image stream, flow cytometric analyses of superoxide production, unbiased metabolomics analyses to assess the downstream consequences on the metabolome, and unbiased transcriptional analyses. Due to the technical complexity, costs and time consumption of these assessments, metabolic dysfunction, and transcriptional analyses will be assessed for QFS and CFS/ME participants and matched healthy controls.

Hair samples will be used to evaluate cortisol and possibly cortisone levels in QFS, CFS/ME, and fatigued JIA participants and healthy controls. Stool and saliva samples will be collected for microbiome analyses in QFS, CFS/ME, and fatigued JIA participants and healthy controls. The microbiome data will be linked to clinical and patient-reported outcomes to investigate whether we can discriminate between subgroups of chronic fatigue, and to identify which operational taxonomic units are relevant for distinction between the disease groups.16sRNA sequencing will be applied to measure bacterial composition at the genus (possibly species) level. Using this 16s phylogenic profiling method, it is possible to get an overview of the community composition of a bacteria and identify differences between groups and variation within groups. DNA extraction, library preparation, sequencing, and basic annotation will be performed. For library construction and 16S rRNA gene sequencing, DNA extraction will be performed with the QIAamp DNA Stool Mini Kit.

The T2 and T4 samples can ultimately be used for objective 5, in which the relationship between individual improvement differences in “fatigue severity” and subsequent changes in biological profiles will be explored.

## Statistical methods

### Statistical methods for primary and secondary outcomes {20a}

To evaluate on the group level the effectiveness of the patient-tailored PROfeel lifestyle advices against generic dietary advices in reducing “fatigue severity” and improving “quality of life” and “self-efficacy” (objective 1), two statistical tests will be performed. The first test, is a 2 × 2 ANCOVA with “fatigue severity” at T2 as dependent variable, “fatigue severity” at baseline as covariate, and intervention condition as fixed factor. This test is used to compare the reduction in “fatigue severity” for the PROfeel-first group against the dietary-first group. The second test is a repeated measures 2 × 2 ANOVA to test whether the order in which participants received the advices is of relevance. The same tests will be performed for secondary outcomes “quality of life” and “self-efficacy”.

To evaluate the effectiveness of the patient-tailored PROfeel lifestyle advices and generic dietary advices in reducing “fatigue severity” and improving “quality of life” and “self-efficacy” at the individual level (objective 2), the permutation distancing test (PDT) will be used. This test is currently under development (see supplement of 41). This test takes into account autocorrelation, handles missing data by making optimal use of all observations, and is specifically developed for *N*=1 designs with a fixed intervention start.

To explore individual effects on pre-post intervention change in the dynamic networks for the participants who first receive patient-tailored PROfeel lifestyle advices first (objective 3), single-subject residual dynamic structural equation modelling (RDSEM) [40] will be used in Mplus. We will model the pre- and post-intervention dynamic networks in one SEM model [41]. This allows for statistical testing of the differences between the pre- and post-network estimates.

To explore the biological profile of adolescents and young adults with QFS and compare it with participants with CFS/ME and JIA, as well as healthy controls (objective 4), we will use between-group ANOVAs and/or linear regression analyses.

Exploration of the relationship between individual differences in “fatigue severity” improvement and biological profile changes pre-post interventions (objective 5) will be conducted with growth mixture modelling (GMM) [42] in Mplus.

### Interim analyses {21b}

Not applicable, no interim analyses will be conducted.

### Methods for additional analyses (e.g., subgroup analyses) {20b}

Not applicable, no additional analyses will be conducted.

### Methods in analysis to handle protocol non-adherence and any statistical methods to handle missing data {20c}

No missing data is expected regarding questionnaires at study visits (KLIK, Eetscore, FFQ, etc.) unless participants drop out of the study. In case of the latter, data will be analyzed up to the point of drop-out. Missing data in the ESM measurement can be handled in the descriptive and dynamic PROfeel reports, e.g., by means of the PDT, although reliability decreases if ESM compliance <70% (see supplement of 41).

### Plans to give access to the full protocol, participant-level data, and statistical code {31c}

Only researchers directly involved in the project will have access to the full protocol, participant-level data, and statistical code. In case non-recovered participants choose CBT, the treating CBT therapist will have access to the baseline assessment and PROfeel ESM data necessary to determine which treatment modules to provide.

## Oversight and monitoring

### Composition of the coordinating center and trial steering committee {5d}

The Wilhelmina Children’s Hospital, part of UMC Utrecht, is the coordinating center of the present study. The research group running the study day-to-day will meet weekly. Principal investigators will join the meeting monthly. The research group will report study progress to the IRB yearly.

### Composition of the data monitoring committee, its role and reporting structure {21a}

A Data Management Plan (DMP) has been prepared and evaluated by the UMC Utrecht’s data quality management team. Please see section “Data management {19}”.

### Adverse event reporting and harms {22}

This RCT has been appreciated by the IRB as having negligible risk. All adverse events (AE) reported spontaneously by the participant, or observed by the investigator within 2 h of the study visit, will be recorded. All AEs will be followed until they have disappeared, or until a stable situation has been reached. Depending on the adverse event, follow-up may require additional tests or medical procedures as indicated, and/or referral to the general physician or a medical specialist.

All serious adverse events (SAE) resulting in death or life-threatening condition will be reported to the accredited IRB within 7 days of first knowledge, followed by a period of maximally 8 days to complete the initial preliminary report. All other SAEs will be reported within a period of maximum 15 days after the investigator first became aware of the SAE. SAEs need to be followed until the end of study, as defined in the protocol.

### Frequency and plans for auditing trial conduct {23}

The QFS-study will be followed by an internal monitor at UMC Utrecht. The monitor is independent from investigators and sponsor. In accordance with the monitor plan, his or her first visit will take place after 3 to 5 inclusions. Afterwards, the monitor will visit annually. During monitor visits, the monitor will inspect the Investigator Site File, informed consents, adherence to study protocol, (S)AE reports, and data safety. Inspection outcomes will be recorded, and required actions and follow-up will be documented in the Monitoring Issues and Action Tracker by the monitor.

### Plans for communicating important protocol amendments to relevant parties (e.g., trial participants, ethical committees) {25}

Amendments are changes made to the research protocol, after the IRB of the University Medical Centre Utrecht has given a positive opinion. The IRB at UMC Utrecht has a standardized procedure for this. The investigator is responsible for submitting an amendment. When an amendment is final, all parties involved will be informed of this by the investigator through written and oral communication.

### Dissemination plans {31a}

The research data will be disclosed unreservedly and reported in accordance with the principles of good clinical practice. Public disclosure will be accomplished by publication of the results in scientific peer-reviewed journals, (poster) presentations during (inter)national conferences, workshops, and the writing of a PhD thesis. Results of this study will be communicated to patient organizations through personal communications, meetings (considering their participation in this project), websites, letters, and/or symposia.

## Discussion

The QFS-study is a comprehensive study examining disrupted biological factors and patient-tailored interventions for fatigued adolescents and young adults with QFS, CFS/ME, or JIA. A few practical or operational issues are expected during the study. The first issue concerns recruitment of QFS participants between the age of 12 and 29 years. As described before, the most recent Q-fever epidemic took place in the Netherlands from 2007 to 2010 [4]. Around that time, the potential youngest participant would have been born. With only a dozen new cases of Q-Fever in The Netherlands per year approximately [5, 6], chances are slim to recruit many young QFS participants. Most QFS participants are expected to be at least 20 years of age, which will be older than most CFS/ME and JIA participants. Not feeling able to balance study or work life with the QFS-study might be a reason for older participants not to participate or to drop-out from the study.

The second issue concerns ESM measurement. During two periods of the study, participants complete ESM measurements approximately five times per day for 4 weeks. Participants might encounter motivation or technical issues during the ESM measurements. The investigator tracks the progress of all participants and takes actions if necessary. To prevent or resolve motivational issues, the investigator explains to the participant why they need to complete the ESM measurement and how many completed surveys are still required. Technical issues with the Ethica app are resolved with the Ethica support team. In case a participant is not able to complete ESM measurement sufficiently due to technical issues, it is possible to restart the ESM measurement period.

The third issue revolves around the behavioral changes imposed in the patient-tailored PROfeel lifestyle and generic dietary advices. The aim of the advices is to give participants insight and autonomy. Hence, the participants act on their advice independently for 12 weeks (unless it is specifically part of the advice to seek guidance). Considering that behavioral change is difficult to impose, the RCT might actually examine which participants are able to independently change behavior and which are not—rather than measuring the effectiveness of the advices itself. The implemented *N*=1 design will be a strength of the study in this aspect.

The last issue concerns healthy control recruitment. We aim to recruit up to 80 healthy controls. We foresee difficulty in finding enough healthy adolescents and young adults willing to participate, especially if they are not familiar with Q-fever and QFS. Therefore, we aim to recruit as many siblings and friends of QFS participants as possible. This strategy might also increase the likelihood of recruiting healthy controls with seropositive *Coxiella burnetii* status. If needed, we will also recruit as many siblings and friends of CFS/ME and JIA-participants as possible and share calls for participation online. We aim to recruit up to 80 healthy controls, to facilitate matching for age and gender in the biological objectives of the QFS-study.

Ultimately, the QFS-study aims to contribute to unraveling disrupted biological factors in QFS and further development of (personalized) treatments for chronic fatigue in adolescents and young adults.

## Trial status

The QFS-study has been described according to version 6 of the study protocol, approved by the IRB of UMC Utrecht on March 25, 2021. The first version of the study protocol received approval from the IRB on June 16, 2020, after which recruitment began in January 2021. Data collection is expected to be completed in December 2022. The QFS-study has been registered at www.trialregister.nl as Trial NL8789 on July 21, 2020.

## Supplementary Information

**Additional file 1: Figure 1.** Descriptive average build-up of fatigue during the day. Note. Figure 1 shows the average build-up of fatigue during the day as presented to the participant in the PROfeel report. The higher the bar, the more fatigue the participant generally experienced during this hour of day. Averages are based on the completed ESM surveys throughout the first 4 weeks of ESM measurement. In case participants receive dietary advices first and patient-tailored PROfeel lifestyle advices second, the averages are based on the completed ESM surveys throughout the first and second ESM measurement periods.

**Additional file 2: Figure 2.** Dynamic between social factors and symptom severity. Note. Figure 2 shows a dynamic network as presented to the participant in the PROfeel report. The participant chose to monitor “being busy with what others think of me” and “feeling pressure to function” as his social factors throughout ESM measurement. Residual dynamic structural equation modelling showed that when presence of the two social factors increased, severity of symptoms (such as fatigue and pain) increased in the hours to follow.

## Data Availability

Only researchers directly involved in the project are allowed to access the final trial dataset. In case a non-recovered participant chooses CBT as post-trial care, the treating CBT therapist is allowed to access that participant’s baseline assessment and PROfeel ESM data if this is necessary to determine which treatment modules should be provided.

## Abbreviations

QFS: Q-fever fatigue syndrome
CFS/ME: Chronic fatigue syndrome/myalgic encephalomyelitis
JIA: Juvenile idiopathic arthritis
CBT: Cognitive behavioral therapy
ESM: Experience sampling methodology
ILD: Intensive longitudinal data
RCT: Randomized controlled trial
UMC Utrecht: University Medical Center Utrecht
CIS: Checklist Individual Strength
CDC: Centers for Disease Control
JADAS: Juvenile Arthritis Disease Activity Score
NNC: The Netherlands Nutrition Centre
VAS: Visual analogue scale
ECCF: Expert Centre for Chronic Fatigue
UU: Utrecht University
IRB: Institutional Review Board
SD: Standard deviation
PedsQL GCS: Pediatric Quality of Life Inventory Generic Core Scale
SES-28: Self-Efficacy Scale-28
PedsQL MFS: Pediatric Quality of Life Inventory Multidimensional Fatigue Scale
RCADS: Revised Child Anxiety and Depression Scale
FFQ: Food Frequency Questionnaire
DMP: Data Management Plan
FAIR: Findable, Accessible, Interoperable, Reusable
GDPR: General Data Protection Regulation
PDT: Permutation distancing test
RDSEM: Residual dynamic structural equation modelling
GMM: Growth mixture modelling
(S)AE: (Serious) adverse event
