# Supplementary figures and images for: Identifying disrupted biological factors and patient-tailored interventions for chronic fatigue in adolescents and young adults with Q-Fever Fatigue Syndrome, Chronic Fatigue Syndrome and Juvenile Idiopathic Arthritis (QFS-study): study protocol for a randomized controlled trial with single-subject experimental case series design
Source: Trials. 2022 Aug 19;23:683. doi: 10.1186/s13063-022-06620-2 (PMC9389501; doi:10.1186/s13063-022-06620-2)

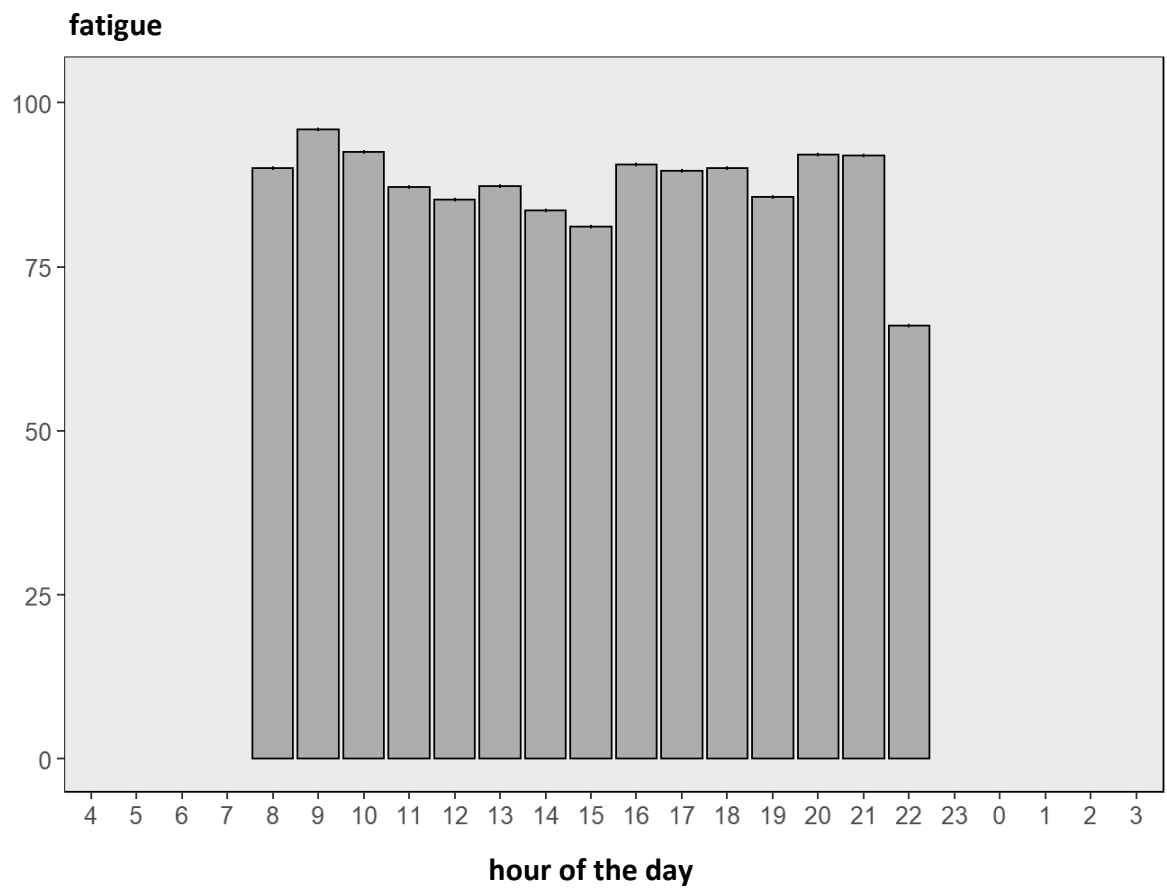

Supplement: Supplementary file 1 — Additional file 1: Figure 1. Descriptive average build-up of fatigue during the day. Note. Figure 1 shows the average build-up of fatigue during the day as presented to the participant in the PROfeel report. The higher the bar, the more fatigue the participant generally experienced during this hour of day. Averages are based on the completed ESM surveys throughout the first 4 weeks of ESM measurement. In case participants receive dietary advices first and patient-tailored PROfeel lifestyle advices second, the averages are based on the completed ESM surveys throughout the first and second ESM measurement periods. [file 13063_2022_6620_MOESM1_ESM.pdf]

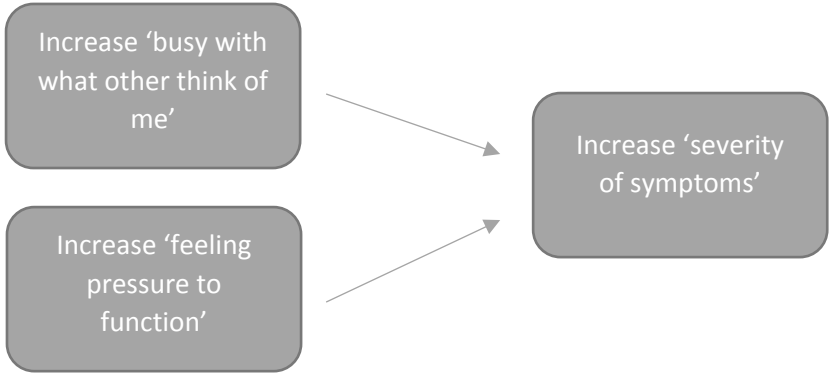

Supplement: Supplementary file 2 — Additional file 2: Figure 2. Dynamic between social factors and symptom severity. Note. Figure 2 shows a dynamic network as presented to the participant in the PROfeel report. The participant chose to monitor “being busy with what others think of me” and “feeling pressure to function” as his social factors throughout ESM measurement. Residual dynamic structural equation modelling showed that when presence of the two social factors increased, severity of symptoms (such as fatigue and pain) increased in the hours to follow. [file 13063_2022_6620_MOESM2_ESM.pdf]
